# Concentrated transdiagnostic and cross-disciplinary micro-choice based group treatment for patients with depression and with anxiety leads to lasting improvements after 12 months: a pilot study

**DOI:** 10.1186/s12888-024-05786-0

**Published:** 2024-05-14

**Authors:** Ane Wilhelmsen-Langeland, Tore Børtveit, Marte Jürgensen, Eirik Søfteland, Sigurd William Hystad, Gerd Kvale

**Affiliations:** 1 Division of Psychiatry, Haukeland University Hospital, Bergen, Norway; 2 Helse i Hardanger, Øystese, Norway; 3 Division of Mental Health and Addiction, Vestfold Hospital, Vestfold, Norway; 4 Department of Medicine, Haukeland University Hospital, Bergen, Norway; 5 Department of Clinical Science, University of Bergen, Bergen, Norway; 6 Department of Psychosocial Science, University of Bergen, Bergen, Norway; 7 Department of Health and Caring Sciences, Western Norway University of Applied Sciences, Bergen, Norway; 8 Department of Clinical Psychology, University of Bergen, Bergen, Norway

**Keywords:** Depression, Anxiety, Concentrated, Micro-choice, Group treatment, Interdisciplinary

## Abstract

**Background:**

A concentrated transdiagnostic and micro choice-based group treatment for patients with depression and anxiety has previously shown to yield significant reduction in symptoms and increased level of functioning from pre to 3-month follow-up. In the present study, we report the results after 12 months follow-up.

**Methods:**

This was a non-randomized clinical intervention pilot study, conducted in line with a published protocol. Sixty-seven consecutively referred patients, aged 19–47 (mean age 32.5, *SD* = 8.0) were included and completed treatment. All had a severity of their problems that entitled them to care in the specialist public mental health care. Self-reported age at onset of symptoms was 17.6 (*SD* = 7.9) years. Mean number of prior treatment courses was 3.5 (*SD =* 3.3; range 0–20). The main objective was to assess the treatment effectiveness by questionnaires measuring relevant symptoms at pre-treatment, 7 days-, 3 months-, 6 months- and at 12-months follow-up.

**Results:**

Validated measures of functional impairment (WSAS), depression (PHQ9), anxiety (GAD7), worry (PSWQ), fatigue (CFQ), insomnia (BIS) and illness perception (BIPQ) improved significantly (*p* < .0005) from before treatment to 12 months follow-up, yielding mostly large to extremely large effect sizes (0.89–3.68), whereas some moderate (0.60–0.76). After 12 months, 74% report an overall improvement in problems related to anxiety and depression. Utilization of specialist, public and private mental health care was reported as nonexistent or had decreased for 70% of the patients at 12-month follow up.

**Conclusions:**

The concentrated, micro-choice based group treatment approach yielded a highly clinically significant reduction in a wide range of symptoms already one week after treatment, and the positive results persisted at 12-month follow-up.

**Trial registration:**

ClinicalTrials.gov Identifier: NCT05234281, first posted date 10/02/2022.

## Background

Anxiety and depression disorders are highly prevalent and represent a huge individual and societal burden [1, 2]. Despite recent systematic reviews showing that evidence-supported treatments are highly effective, also when delivered as part of routine clinical care, reports on long-term effects are scarce [3–5]. Based upon a novel concentrated group treatment format, we previously demonstrated beneficial effects on level of functioning as well as primary symptoms three months after the intervention [6]. We also showed that the treatment was highly acceptable, with clinically significant improvement in functional impairment, reduction in anxiety and reduction in depression [6].

In the current paper we report on the results after 12 months of follow-up, aiming to explore potential improvements in level of functioning, levels of anxiety, depression, insomnia, fatigue and worry at multiple follow-up time-points. Further, we aimed to evaluate changes in illness perception. We also explored the participants’ utilization of health care, potential changes in psychotropic medication, overall evaluation of potential change regarding symptoms of anxiety and depression and evaluation of to what extent they were able to maintain changes they initiated during or before the concentrated treatment, all measured by self-report.

### Hypotheses

Based on our experiences with this particular and other concentrated treatment formats [6–11], we expected a significant improvement in level of functioning and clinically relevant effects on symptoms of anxiety and depression, as well as significant reduction in symptoms of worry and fatigue, and significant improvement of illness perception. We expected that these improvements would be clinically relevant 12 months after treatment. Based on experiences from concentrated treatment for OCD, we did not expect a significant rapid reduction in insomnia symptoms [12]. We expected utilization of health care to be unchanged or reduced at all follow-up points and that the improvement would be maintained at a clinically relevant level after 12 months.

## Methods

The methods have been described in greater detail in the protocol paper as well as in the 3-month follow-up paper [6, 14]. This pilot study is part of the “Project Development of Smarter Health Solutions” (PUSH project), a collaboration between Haukeland University Hospital (Bergen, Norway) and Helse i Hardanger (HiH; Kvam, Norway). HiH delivers concentrated treatment to patients with chronic low-back pain, diabetes type 2, long COVID without acute improvement, chronic obstructive pulmonary disease condition as well as to patients with chronic anxiety and depression [6, 13, 14]. The first trans-diagnostic experiences with this group rehabilitation showed that treatment satisfaction was high across all illness groups (low back pain, long COVID and type 2 diabetes), functionality was improved, and illness perception had positively changed [15]. The main architects behind the content of the concentrated micro-choice based program for patients with depression and/or anxiety are the second and last authors of the current article (TB and GK).

### Referral procedures

General practitioners in the uptake area were encouraged to refer patients to the project. The patients were screened for participation in the project via a short, structured telephone-interview within 10 days after referral. Patients with symptoms of a severity which granted them right to treatment as a part of specialist public health care according to the Norwegian priority guidelines, were offered further inclusion [16]. They then had two consultations with a clinical psychologist, either face-to-face or via video on a secure online platform. Most patients had the first consultation within 4 weeks from referral. Timeslots for implementation of groups were planned to fit with the schedule of the group leaders, and eligible patients were offered participation successively upon availability. Waitlist (1–10 weeks) were in line with the priority guidelines in Norway.

### Patients

#### Inclusion and exclusion criteria

Inclusion and exclusion criteria were the same as for the 3-month pilot study [6]; patients between 18 and 47 years of age, which leaves a minimum of 20 years in the work force according to Norwegian retirement age. Patients were eligible for inclusion if they fulfilled the ICD-10 [17] criteria for one of the following disorders: depression (F33.1; *n* = 33 (49.3%)), F33.2; *n* = 1 (1.5%)); generalized anxiety disorder (F41.1; 7 (10.4%)); mixed anxiety and depressive disorder (F41.2; *n* = 16 (23.9%)); other mixed anxiety disorders (F41.3; *n* = 9 (13.4%)) or unspecified anxiety disorder (F 41.9; *n* = 1 (1.5%)), and this being their main psychiatric problem. Patients with a principal diagnosis of obsessive-compulsive disorder (F42), panic disorder with or without agoraphobia (F41.0/F40.0), social anxiety disorder (F40.1) or chronic fatigue (G93.3 or F48.0) were excluded since patients with these disorders already had adequate concentrated treatment opportunities within the catchment area. Single depressive episodes were not excluded per se, but all patients had a chronicity in their problems and no patients with a single depressive episode were referred to this treatment. F41.2 was included as it is a common diagnosis that may lead to a full syndromal psychiatric disorder [18], which may be avoided with targeted treatment.

The patients went through a diagnostic interview by a trained psychologist using the Mini International Neuropsychiatric Interview (M.I.N.I.; [19]). M.I.N.I is a short structured diagnostic interview, which screens axis-1 DSM-IV disorders [20]. For the F41.2 diagnoses we used chapter Z. of the M.I.N.I. PLUS [21].The participants were fluent in Norwegian.

Exclusion criteria were bipolar disorder, psychosis, ongoing severe or primary substance abuse/dependence, mental retardation based on previous medical history, very low BMI in need of medical attention, and ongoing suicidal ideation. Also, if the patients had a physical condition which prevented them from participating in physical exercise, they were not offered participation.

**Fig. 1:**
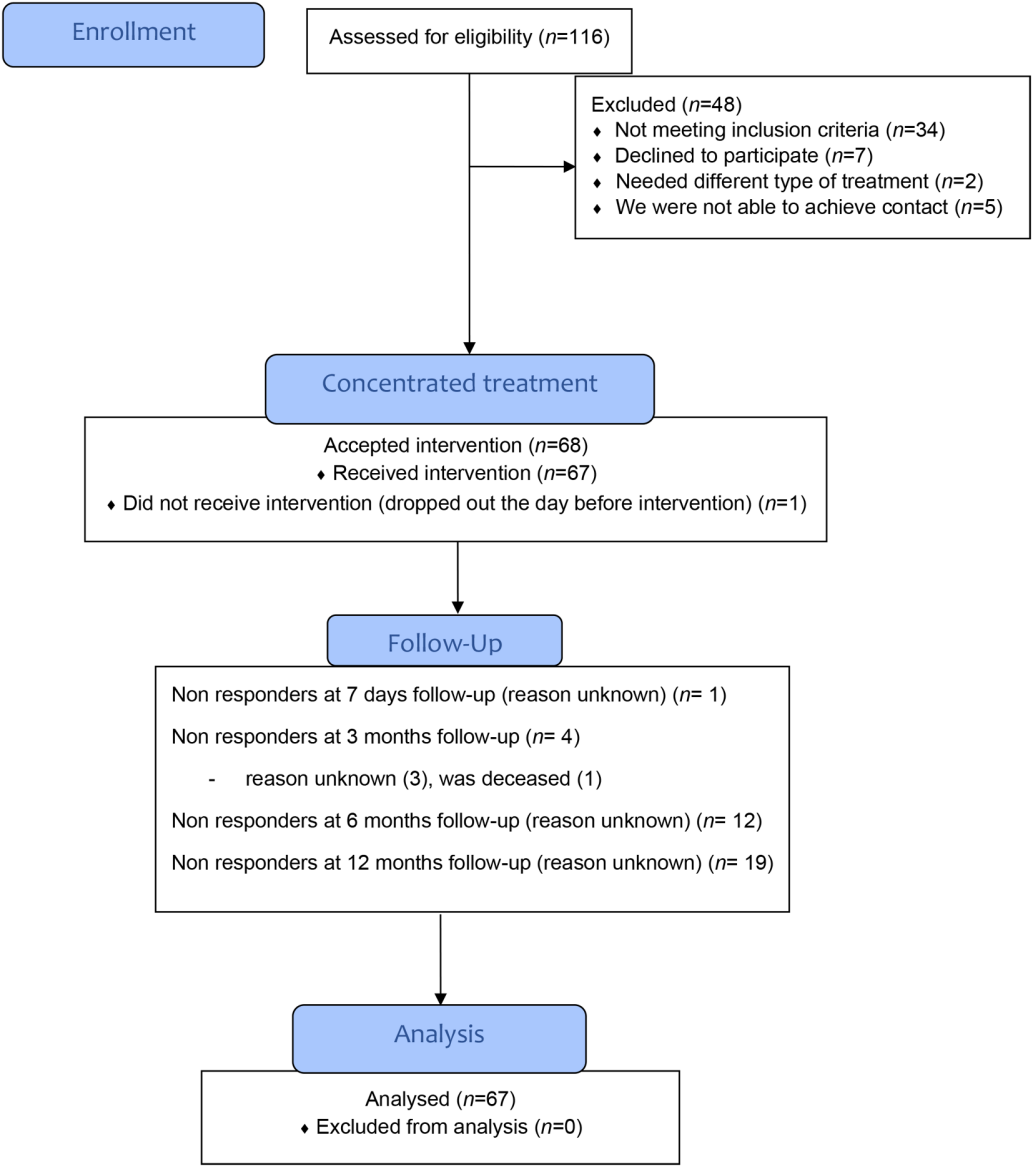
Flow-chart showing patient flow through the project

During the inclusion period, 116 patients were referred and 34 were excluded as they did not fulfil the inclusion criteria. Seven declined to participate as they were ambivalent about the concentrated group format, and were not further evaluated, two needed a different type of treatment with longer follow-up, and finally we were unable to get in contact with five of the referred patients (see Fig. 1 for flow-chart).

Table 1 displays a summary of background data for the sample. In total 91.8% of the patients that had answered the relevant questions, had received previous treatment (specialist health care, primary/community health care, private sector) for the current disorders. The majority of the patients (52.7%) reported that they used prescribed medication for depression and/or anxiety. We did not initiate changes of medication.

**Table 1:** Background information on patient sample (*n* = 67)

	M (SD)	*n*	*n* (%)
**Gender**			
Female		37	55.2
**Age**	32.5 (7.95)		
**Age at onset of problems**	17.6 (7.91)		
Duration of problems	14.68 (9.39)		
Previous treatment courses*	3.45 (3.34)		
None		4	6
One		10	14.9
Two		9	13.4
Three or more		26	38.9
Medicated for anxiety/depression* Yes		34	50.7
Marital status*			
Single		34	50.7
Married or cohabitating		32	47.8
Unknown		1	1.5
**Daily responsibility for children**			
Yes		25	37.3
**Educational status**			
Junior high school		3	4.5
High school		32	47.8
College/university		22	32.8
Apprenticeship		10	14.9
**Work status**			
Employed		52	77.6
Unemployed		6	9
Job applicant		1	1.5
College student		2	3
High school student		2	3
Unknown/other		4	6
Months out of work*	10.62 (19.57)		
**Welfare benefits**			
Avg. percentage of social welfare received¤	52.61 (46.66)		
Not on welfare		26	38.8
On sick leave from work		20	29.9
Between sick leave and disable benefit (AAP)		15	22.4
Disable benefit		4	6
Cared for by family		2	3

*Note* *missing info regarding previous treatment courses from eighteen participants, missing info from two participants regarding medication for anxiety/depression, missing info from one participant regarding marital status, missing info from six participants regarding months out of work

¤ Percentage of social welfare refers to what degree of social welfare the patients received as opposed to salary from work. The number refers to that 52.6% of the patients` total income is received from welfare benefits; sick leave, AAP or disable benefit

### Ethics

Written consent was signed by all participants before data collection. The project was approved by the ethics committee in Western Norway (REK West 2020/101.638) and was conducted in accordance with the Helsinki declaration. ClinicalTrials.gov Identifier: NCT05234281, first posted date 10/02/2022.

### Assessment

#### Questionnaires

Patients answered standardized self-report questionnaires online via a secure platform before treatment and at 7 days and 3-, 6- and 12-month follow up. If patients did not complete self-report questionnaires according to a pre-set time limit, an automated reminder text message was sent to their phones.

**Functional level** was assessed pre-treatment and at 3-, 6- and 12-month follow-up by Work and Social Adjustment Scale (WSAS; [22]) which is a short questionnaire measuring the impact of the disorder on aspects of work and social activities. The scale consists of five items rated from 0 (not at all) to 8 (very severe), and a higher score indicates higher impairment (the maximum score is 40). The cut-off for moderately severe or worse psychopathology is > 20, scores between 10 and 20 are associated with significant functional impairment but less severe clinical symptomatology, and scores < 10 are associated with subclinical populations. The scale has good psychometric properties [22]. Cronbach’s α in the current study were 0.73 at baseline, 0.91 at 3-months follow-up, 0.93 at 6-months follow up and 0.91 at 12-months follow-up.

**Depressive symptoms** were measured at pre-treatment, post-treatment and at 3-, 6- and 12-month follow-up, by the Patient Health Questionnaire (PHQ-9; [23]). The PHQ-9 is a self-report scale with nine items (0 to 3 scale). The maximum score is 27. A score of 10 or more is indicative of a depressive disorder [23]. Recently, the cut-off value was suggested to be 14 or higher [24]. Cronbach’s α in the current study were 0.75 at baseline, 0.85 at post-treatment, 0.88 at 3-months follow-up, 0.90 at 6-months follow up and 0.90 at 12-months follow-up.

**Symptoms of generalized anxiety** were measured at pre-treatment, post-treatment and at 3-, 6- and 12 month follow-up by the Generalized Anxiety Disorder scale (GAD-7; [25]). The GAD-7 is a self-report scale with seven items (0 to 3 scale). The maximum score is 21 and the suggested cut-off of is 10 points. Cronbach’s α in the current study were 0.75 at baseline, 0.85 at post-treatment, 0.88 at 3-months follow-up, 0.90 at 6-months follow up and 0.90 at 12-months follow-up.

**Worry and rumination** were measured pre-treatment, post-treatment and at 3-, 6- and 12 month follow-up by Penn State Worry Questionnaire (PSWQ; [26]). PSWQ was added as part of the data collection after enrollment of 19 patients pre, and after enrollment of 10 patients post 7 days and at 3-month follow-up. 48 patients received this questionnaire at pretreatment, all participants received the questionnaire at 6 months follow-up, and it was added at 12 months after enrollment of 9 patients, hence all patients received this questionnaire 6 months after treatment, while 57 received it also at 7 days after treatment and 3- and 12- months follow-up. The PSWQ is a self-report scale with 16 items (on a Likert scale; 1=“not at all typical”, 5=”very typical of me”), possible range of the total score is 16–80, where a higher score indicates more worry. Cronbach’s α in the current study were 0.89 at baseline, 0.92 at post-treatment, 0.91 at 3-months follow-up, 0.91 at 6-months follow up and 0.94 at 12-months follow-up.

**Symptoms of fatigue** were measured by Chalder Fatigue Questionnaire (CFS; [27]). The scale consists of 11 items and is scored on a 4-point Likert scale (0 = less than usual, 1 = no more than usual, 2 = more than usual, 3 = much more than usual and a bimodal scale where 0 and 1 yields 0 on bimodal score and 3 and 4 yields 1 in bimodal score. The CFQ can provide a sum score (range 0–33) or scores for two components: one that measures physical fatigue (questions 1–7) and one that measures mental fatigue (questions 8–11). The average score based on a normative study from Norway is 12.2 (*SD* 3.9) [28]. Cronbach’s α in the current study were 0.88 at baseline, 0.83 at post-treatment, 0.87 at 3-months follow-up, 0.94 at 6-months follow up and 0.94 at 12-months follow-up.

**Insomnia symptoms** were measured pre-treatment, post-treatment and at 3-, 6- and 12 month follow-up by the Bergen Insomnia Scale (BIS; [29]). The BIS is developed based on symptoms-related questions according to the American Psychiatric Association Diagnostic and Statistical Manual of Mental Disorders-IV-text revision (DSM-IV-TR; [30]). It is a 6-item questionnaire, which is scored on an 8-point Likert scale referring to the number of days a week for which a specific symptom is experienced (0–7 days). The total score ranges from 0–42. An algorithm of i) scoring 3 or more on at least one of items 1–4, ii) and 3 or more on at least one of items 5 and 6, is considered as ”caseness” of having insomnia. Cronbach’s α in the current study were 0.77 at baseline, 0.83 at post-treatment, 0.73 at 3-months follow-up, 0.84 at 6-months follow up and 0.83 at 12-months follow-up.

**Illness perception** was measured at pre-treatment, post-treatment and at 3-, 6- and 12month follow-up by the Brief Illness Perception Questionnaire (BIPQ), which is a 9-item questionnaire designed to assess cognitive and emotional representations of illness [31]. BIPQ was added as part of the data collection after enrollment of 24 patients after 3 months and 12 months, hence all patients received this questionnaire pre and 6 months after treatment, while 42 received it also at 3- and 12-months follow-up. A higher BIPQ score indicates a greater perceived psychological burden of illness. Questions are graded from 0 to 10. The last item deals with the perceived cause of illness, in which respondents list the perceived three most important causal factors in their illness. The scale has good psychometric properties [32]. Cronbach’s α in the current study were 0.30 at baseline, 0.88 at post-treatment, 0.91 at 3-months follow-up, 0.91 at 6-months follow up and 0.89 at 12-months follow-up.

**Utilization of health care.** We also recorded the patient-reported utilization of health care, by the following question asked three months, six months and twelve months after the concentrated treatment: “Compared to before the treatment was initiated, how has your utilization of health services directly related to anxiety and depression been?” Examples of health services listed were specialist health care, community services, private health services, and general practitioner. Answers could be (a) no use, (b) less use than before, (c) unchanged, and (d) more use than before.

**Changes in psychotropic medication.** Three, six and twelve months after treatment, the patients answered the following question regarding use of psychotropic medication: “Compared to the start of the treatment, how has your use of medication related to anxiety and depression been?” Answers could be (a) less use, (b) unchanged, (c) more and (d) not applicable [6].

**Clinically relevant evaluation of change.** Since our sample is composed of patients with several depression/anxiety disorders, neither PHQ-9 nor GAD-7 would be relevant for all patients. Also, due to limited number of participants, further sub-group analyses were not feasible [6]. However, we included a question addressing overall change in their relevant symptoms. The question was tested out on a smaller sample of patients who reported that they had no problems understanding and assessing this [6]. Thus, three, six and twelve months after treatment, the patients reported on the following questions: 1. “In all, how would you evaluate your anxiety and depression now, compared to before the treatment?” Answers could be (a) substantial improvement, (b) improvement, (c) minimal improvement, (d) no change, (e) minimal deterioration, (f) deterioration, (g) substantial deterioration [6]. We have collapsed a-b to “Improvement”, c-e to “No change” and f-g to “Deterioration” in the results section.

**Maintenance of change.** Three, six and twelve months after treatment, the patients answered the following question regarding whether or not they were able to maintain changes regarding daily routine and principles that were practiced during the concentrated treatment: (A) To what extent have you kept the wake-up time you had decided on?; (B) To what extent did you keep up the frequency and intensity of physical activity that you decided on?; (C) To what extent did you let your symptoms control your life? And finally (D) To what extent did you practice what you learned during the concentrated treatment [33]. Answers were to be graded from 0 to 100.

### Procedure

The treatment was given in three phases: (1) The preparation phase, (2) The concentrated micro-choice based treatment and (3) Maintenance and implementing the change into everyday life. If the patient wanted to initiate treatment at the end of phase 1, informed consent was signed. One week prior to treatment, the leader of the group called each patient to ensure they had received all necessary information and were ready. During the concentrated treatment (four days + an introductory meeting the night before) the patients were discouraged from using anxiolytics and alcohol.

### Treatment

The concentrated treatment took place between 2020 and 2022 at an outpatient clinic outside Bergen, Helse i Hardanger, and the participants were accommodated at a hotel in the same building Monday through Friday. The treatment was delivered to groups of 5–9 patients. The outline and content of the treatment is described elsewhere [14] and the treatment for this particular group of patients is further described in the published paper with 3-month follow-up data for 42 patients [6].

### Data completion

A total of 67 patients are included in the dataset. Of the 67 study participants, all but one completed the post-treatment questionnaires one week after treatment (98.5%), 64 (94%) at 3-month follow-up, 55 (82%) at 6-month follow up and 48 (72%) at 1 year follow-up. We checked, but found no significant baseline differences between the completers compared to those lost to follow-up.

### Statistical analyses

Mixed-effects regression models were used to compare pre-treatment scores on WSAS, GAD-7, PHQ-9, PSWQ, CFQ, BIS and BIPQ with scores at later assessment points (post 7 days (not WSAS), post 3 months, post 6 months and post 12 months follow-up). Effect sizes of change over time were calculated using Glass´ Δ, with estimated means from the mixed regressions used in the numerator and pre-treatment *SD* in the denominator. Glass’s Δ is the recommended effect size for intervention studies in which there are reasons to believe that the treatment will influence the standard deviation as well as the mean [34]. An effect size is commonly interpreted as small (0.2), moderate (0.5), and large (0.8).

Changes in caseness of insomnia over time were examined with a Cochran’s Q test for overall differences between timepoints and followed up with McNemar’s tests with Bonferroni corrections comparing baseline to post, 3 months, 6 months, and 12 months. Missing values in these analyses were handled by last observation carried forward (LOCF). All analyses were conducted using Stata version 17.0 [35].

## Results

### Attrition

All but one (98.5%) of the included patients started the treatment, and all who started the concentrated treatment (met the first day), completed the treatment, hence we had 0% attrition.

### Clinical outcomes

**Fig. 2:**
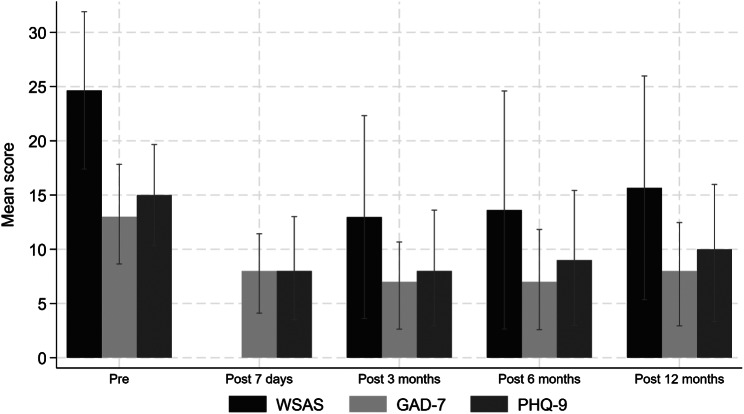
Bar chart showing mean scores in problems relating to work and social adjustment, symptoms of anxiety and symptoms of depression at different time points

Results from the mixed-effects regressions showed that patients had statistically significant improvements in all clinical outcomes from pre-treatment to post-treatment (see Table 2; Fig. 2). All improvements were maintained at 3 months, 6 months, and 12 months follow-up. All improvements can be interpreted as large judging by the effect sizes presented in Table 3, except for the improvements in PSWQ from pre- to post-treatment and 12 months follow-up, and the improvements in BIS from pre- to post-treatment, 6 months follow-up, and 12 months follow-up, which can be interpreted as medium.

**Table 2:** Estimated marginal means (SE) from mixed linear regression and pairwise comparisons between pre- and post-measures

	Pre		Post 7 days	*p*	3 mos. - FU	*p*	6 mos. - FU	*p*	12 mos. - FU	*p*
WSAS	24.64 (1.13)	vs.			12.95 (1.16)	< 0.001	13.81 (1.21)	< 0.001	15.49 (1.27)	< 0.001
GAD-7	13.34 (0.53)	vs.	7.77 (0.53)	< 0.001	6.65 (0.54)	< 0.001	7.30 (0.57)	< 0.001	7.74 (0.59)	< 0.001
PHQ-9	15.01 (0.65)	vs.	8.21 (0.66)	< 0.001	8.10 (0.67)	< 0.001	9.11 (0.69)	< 0.001	9.47 (0.72)	< 0.001
PSWQ	62.34 (1.58)	vs.	55.53 (1.52)	< 0.001	51.03 (1.54)	< 0.001	50.85 (1.52)	< 0.001	52.48 (1.64)	< 0.001
CFQ	20.42 (0.77)	vs.	15.28 (0.77)	< 0.001	12.34 (0.78)	< 0.001	12.73 (0.81)	< 0.001	15.43 (0.89)	< 0.001
BIS	22.82 (1.10)	vs.	17.19 (1.10)	< 0.001	14.50 (1.12)	< 0.001	16.25 (1.16)	< 0.001	15.69 (1.23)	< 0.001
BIPQ	51.25 (1.50)	vs.	34.01 (1.79)	< 0.001	30.39 (1.84)	< 0.001	30.45 (1.59)	< 0.001	35.21 (2.09)	< 0.001

*Note* WSAS = Work and social adjustment scale (range 0–40); GAD-7 = General anxiety disorder (range 0–27); PHQ-9 = Patient health questionnaire (range 0–21); PSWQ = Penn State worry questionnaire (range 16–80); CFQ = Chalder fatigue Scale (range 0–33); BIPQ = Brief illness perception questionnaire (range 0–80); BIS = Bergen Insomnia Scale (range 0–42), FU = Follow-up

Figure 2. Mean scores on WSAS, GAD-7, and PHQ-9 across five measurement points. Error bars represent one standard deviation above/below the mean. WSAS = Work and social adjustment scale (range 0–40); GAD-7 = General anxiety disorder (range 0–27); PHQ-9 = Patient health questionnaire (range 0–21). WSAS was not measured at 7-day follow up.

**Table 3:** Effect Sizes of Improvements from Pre-Treatment to Post-Treatment, 3 Months Follow-up, 6 Months Follow-up, and 12 Months Follow-up

	Post 7 days	3 mos. - FU	6 mos. - FU	12 mos. - FU
WSAS	—	1.61	1.49	1.26
GAD-7	1.19	1.43	1.29	1.20
PHQ-9	1.46	1.49	1.27	1.19
PSWQ	0.60	1.01	1.03	0.88
CFQ	0.98	1.54	1.47	0.95
BIS	0.64	0.94	0.75	0.81
BIPQ	3.02	3.67	3.66	2.81

*Note* WSAS = Work and social adjustment scale; GAD-7 = General anxiety disorder;

PHQ-9 = Patient health questionnaire; PSWQ = Penn State worry questionnaire;

CFQ = Chalder fatigue Scale; BIPQ = Brief illness perception questionnaire; FU = Follow-up.

Effect sizes computed as Glass’s Δ = $\frac{{M}_{pre}-{M}_{post}}{{SD}_{pre}}$

### Caseness of insomnia

Caseness criteria of insomnia were fulfilled by 88.1% of patients at treatment start (Table 4). Exact McNemar’s tests showed that the proportion of caseness decreased statistically significantly at every time-point except at 6 months follow-up.

**Table 4:** Percentages of Participants Fulfilling Caseness Criteria of Insomnia and Comparisons Between Timepoints

	Timepoint	Insomnia caseness		Comparison	χ^2^	*p* ^a^
1	Baseline	88.1% (*n* = 59)		—	—	—
2	Post	62.7% (*n* = 42)		1 vs. 2	15.21	< 0.001
3	3 mos. - FU	55.2 (*n* = 37)		1 vs. 3	18.62	< 0.001
4	6 mos. - FU	71.6 (*n* = 48)		1 vs. 4	7.12	0.051
5	12 mos. - FU	59.7 (*n* = 40)		1 vs. 5	15.70	< 0.001

*Note* Cochran’s χ^2^ (4) = 27.28, *p* < .001

^a^ McNemar’s exact significance probability with Bonferroni correction for multiple testing (*p* × m)

### Utilization of health care

On the question: “Compared to before the treatment was initiated, how has your utilization of health services directly related to anxiety and depression been?”, most patients reported “not using” or “less use” than before regarding specialist health care, community services and private health services (for details see Table 5). Most of the participants reported that they had not seen or had seen their GP less than before.

**Table 5:** Utilization of health care at 3, 6 and 12-month follow-up, *n* = 67

	No use	Less than before	No change	More than before
At 3-month follow-up*				
*Specialist health care*	79.4%	7.9%	9.5%	3.2%
*Community services*	79.4%	6.3%	12.7%	1.6%
*Private health care*	87.1%	3.2%	9.7%	0%
*General practitioner*	36.5%	34.9%	23.8%	4.8%
At 6-month follow-up*				
*Specialist health care*	74.6%	6.3%	12.7%	6.3%
*Community services*	79%	4.8%	12.9%	3.2%
*Private health care*	90.3%	0%	6.5%	3.2%
*General practitioner*	29%	38.7%	25.8%	6.5%
At 12-month follow-up*				
*Specialist health care*	58.7%	15.9%	22.2%	3.2%
*Community services*	38.8%	14.9%	16.4%	1.5%
*Private health care*	79.2%	4.8%	12.9%	3.2%
*General practitioner*	19.4%	41.9%	30.6%	8.1%

*Note* Percent is given in valid percent

### Change in psychotropic medication

Before treatment, 52.3% reported using medication for anxiety and depression. At 3 months follow-up 25.8% reported less use, 8.1% reported more use, 32.3% reported unchanged use of such medication. At 6 months follow-up 17.5% reported less use, 7.9% reported more use, 32.3% reported unchanged use of such medication. At 12 months follow-up 19% reported less use, 15.9% reported more use, 34.9% reported unchanged use of such medication.

### Maintenance of change

The participants reported to what extent they had kept the rise time (the time they got out of bed) they had decided on, at 7 days-, 3 months-, 6 months- and 12 month follow up. The mean answers were 84%, 75%, 71% and 67%, respectively. When it comes to the frequency and intensity of physical activity that they decided on, the mean answers to what extent they had kept their planned level were 78% at 7 days, 72% at 3 months, 67% at 6 months and 66% at 12 months follow-up. Regarding to what extent they had let their symptoms control their life, the mean answers were 28% at 7 days, 33% at 3 months follow-up, 34% at 6 months follow-up and 44% at 12-month follow-up. Finally, “to what extent did you practice what you learned during the concentrated treatment”, the mean answers were 84%, 80%, 79% and 68% at 7 days, 3 months, 6 months, and 12 months follow-up respectively.

### Overall change in problems related to anxiety and depression

Overall, 84% of the patients reported “improvement” at 3-month follow-up, 77% at 6-months follow-up and 74% at 12-months follow-up (see Table 6).

**Table 6:** Evaluation of overall change regarding problems with anxiety and depression

*All in all, how would you consider your problems related to anxiety and depression?*	At 3-month follow-up	At 6-month follow-up	At 12-month follow-up
	*n*(%)	*n*(%)	*n*(%)
Improvement	53 (84.1)	49 (77.8)	46 (73)
No change	8 (12.7)	14 (22.2)	16 (22.8)
Deterioration	2 (3.2)	0 (0)	1 (1.6)

*Note* Percent is given in valid percent

## Discussion

The patients included in this pilot study had a mean duration of their anxiety/depression symptoms of more than 14 years, 88% had likely insomnia according to BIS, 67.2% had received previous unsuccessful courses of treatment, 50.7% used medication for their anxiety/depression symptoms, and 58.3% had some type of disability pension or sick leave due to their disorders. As hypothesized, the significant effects at 7-day follow-up were maintained after 12 months with regards to symptoms of functioning, depression, anxiety, worry, fatigue and illness perception. Symptoms of anxiety and depression, which were the key targets for the intervention, were below clinical cut-off (< 10 on PHQ-9 and GAD-7) at 12-month follow-up [25, 36].

In our view, to observe a significant reduction in symptoms of anxiety and depression in such a tormented group, amid the COVID-19 pandemic, is quite remarkable. Most reports indicate that symptoms of i.e. anxiety, depression and sleep disorders increased during the same time period [37–41], while we show a decrease in a group that had been out of work for an average of 10.6 months, had been through several unsuccessful treatment series in public mental health care. They were severely affected not only by their psychiatric illness, but also by worry, fatigue, unfortunate perceptions about their illness and insomnia symptoms.

### Comparison with other studies

A comparison with the Norwegian Prompt Mental Health Care (PMHC) program [42] showed that the current study had a significantly higher proportion of patients with previous treatments (67.2 vs. 16%), with psychotropic medication (52.9 vs. 22%), being single (50.7 vs. 31%), higher mean GAD-7 score (13.2 vs. 10.1), and higher mean PHQ-9 score (15.1 vs. 12.5) before the start of treatment. This indicates that the current sample was more severely affected than the PMHC-sample. The average score on CFQ in the Norwegian population has been shown to be 12.2 (3.9) [28], while the mean score in our sample before treatment was 20.4 (SD 5.2), meaning our sample was also significantly fatigued. In a recent study, it was found that insomnia symptoms increased after the COVID-19 pandemic and the mean score on the BIS after the increase was 13.5 (SD 10.2) [43], while in our sample the mean score before treatment was 22.8 (SD 8.8), hence they were also severely affected by symptoms of insomnia.

As described in the protocol paper [14], the cross disciplinary intervention lasted four full days with an additional preparatory meeting the night before. The intervention included elements from cognitive behavioral therapy, behavioral activation, ACT, metacognitive approaches as well as brief mindfulness exercises, all focused on breaking patterns of unhelpful emotional regulation, basing change on a systematic micro-choice based approach and introducing an eat-sleep-move pattern compatible with having a job.

The intervention was highly acceptable (> 90%) and levels of satisfaction were high, in accordance with what we reported on at 3-month follow-up [6]. Compared to pre-treatment, clinically meaningful improvements in level of functioning were seen with correspondingly increased perceived understanding of the health challenges. In our view, the swiftness of symptom reduction (measured first at 7 days) was noteworthy, although in line with earlier experiences with the concentrated treatment format [6, 7]. As expected based on previous experience with concentrated treatment for OCD [8], the improvements did not deteriorate between post-treatment (7 days) and follow-up (3, 6 and 12 months). Even though it was expected, we still consider these findings remarkable, as this group of patients were more heterogeneous than previous study samples. Also, the low attrition rate we saw in our study is rare; in a recent meta-analysis on treatment for depressive disorder, the authors report 25,1% average attrition rate and they found no significant difference between individual and group treatment [4].

Large effect sizes were obtained on measures of anxiety (GAD-7), depression (PHQ-9), fatigue (CFQ) and illness perception (BIPQ) lasting for 12 months. Medium to large effect sizes were seen for reduction in worry (PSWQ) and insomnia symptoms at the different measurement points. A recent meta-analysis on cognitive therapy for adults with depressive disorders in routine clinical care, reported effect sizes between 1.51 and 1.85, while the treatment was given over a period of 5–20 weeks [42]. A multicenter randomized controlled trial with three arms; internet-based cognitive behavioral therapy (ICBT), exercise and treatment as usual over 12 weeks, yielded effect sizes of 0.24–0.46 at 12-month follow-up [44]. At 12-month follow-up after up to 30 weeks of cognitive behavioral therapy and acceptance and commitment therapy for adult depression, the researchers report effect sizes of 1.26–1.60 [45]. The Unified Protocol (UP) is a transdiagnostic treatment that targets shared mechanisms in anxiety disorders, and it aims to be a single protocol that can be used for anxiety disorders, instead of multiple single-disorder protocols (SDP) [46]. In a study using UP compared to SDP, effect sizes were small (0.26) for the effect on anxiety after 12 months for both treatment protocols [47]. Hence, our results are superior to some - and comparable to other treatments for depression and anxiety - given over a substantially longer time period.

### Micro-choices as a generic model for change?

The concept of deliberate micro-choices in order to systematically break behavior patterns that perpetuate health problems seems to be an acceptable approach that works well as an analogy for many people. Instead of focusing on symptom reduction, which is out of the patients` control, they learned to act on planned behavior, irrespective of symptoms. This triggered unpleasant symptoms of anxiety and depression, but also a sense of being in control.

At three months follow-up 84.1% reported overall improvement of their problems and this was to a large degree maintained at twelve months after treatment. Adjacent to the escalation in mental problems during the pandemic, the improvement we demonstrate are even more impressive and seems to tell a story of a sturdy treatment approach that gives the recipients an opportunity to take control over symptoms they previously felt they could not control.

### Insomnia symptoms after treatment for anxiety and for depression

Insomnia symptoms have earlier proven to remain even after successful treatment for depression while treatment for insomnia has shown to improve both symptoms of insomnia and depression [48]. We did not expect symptoms of insomnia to decrease rapidly in our patients in line with previous findings from Concentrated Exposure Treatment for OCD [12]. Hence, the rapid reduction in insomnia is a surprising result. The authors of the OCD-study conclude that 4 days might be too short to change insomnia symptoms since the BIS measures insomnia symptoms in the past month and even claim it is unrealistic to expect significant impact on these symptoms after only four days of treatment [12]. In our sample, the caseness of insomnia was higher (88% vs. 81.3% in the OCD study) as well as the average total score (22.82 vs. 17.75 in the OCD study). The rapid significant reduction in insomnia symptoms may be due to a more explicit focus on sleep-wake factors in our intervention than the OCD-study. Our participants also had a 5-minute highly focused talk (either live or from an audio-file) the last day of treatment focusing on sleep regulation and precipitating factors that lead to maintenance of insomnia symptoms, by a somnologist/sleep expert (first author AWL).

On the other hand, although we observed a reduction on insomnia symptoms and in the proportion of patients having indication of insomnia (from 88% before treatment to 59.7% at 12-month follow-up), a large proportion of our participants still had insomnia symptoms and “caseness” 12 months after treatment. Insomnia has been found to be a risk factor for anxiety and depression [49] as well as for sick leave [50], hence we concur with Hagen et al. [12] in that treatment directly addressing insomnia-symptoms might be valuable as a bigger part of concentrated treatment for anxiety and depression in the future.

## Strengths and limitations of the pilot study

### Strengths

The main strength of this study is that the treatment is given over only 4 days, which is less intruding in the patients` everyday life than treatments given over a longer time span. We have shown and discussed that our concentrated treatment over 4 days has rapid and comparable or even superior effect sizes to other treatments for anxiety and depression in adults, given over 8–14 weeks, which is a great strength. Also, a strength is the long-term follow-up of 12 months. Other comparable studies of patients with similar complaints of anxiety and of depression, report attrition rates of 25.1–30.8% [4, 46].

The study shows that patients with long-standing depression and/or anxiety, and with substantial prior treatment experiences, could benefit from a different and more concentrated micro-choice based treatment format and that the improvements are long lasting across a wide array of symptoms known to affect functioning in all aspects of life. Mental health problems are on the rise; hence developing effective treatments is a challenge that the public health care is facing. Our results show a robustness in large effect sizes at long-term follow-up, obtained during the COVID-19 pandemic. Our patient sample was on average 32.5 years of age, meaning they were in a typical age caring for children. Treatments that are short-lasting represent an obvious strength for this age group, as they may have parental obligations at home. Mentally healthy parents will be more capable of raising mentally healthy children [51, 52].

### Limitations

The major limitation of this study is that we have no control group and hence cannot attribute the positive changes to our intervention. Limitations of the study include the moderate sample size, the relatively experienced group leaders meaning generalizability to less experiences group leaders may need to be investigated and that it is not possible to pinpoint what are the effective components of the treatment. Because of the diversity among the participants, sub-group analyses were not regarded as feasible. It is possible that the results are not generalizable to patients who fall outside of the inclusion/exclusion criteria. For example, this might apply to older participants (> 47 years), those with limited digital competency, or patients with other comorbid illnesses than those included in this sample. Conducting a treatment in groups during the Covid pandemic is per se a social event and may possibly have been health-promoting in itself. We cannot rule out this possible bias towards our results. Finally, several of the data were self-reported and not based on validated questionnaires (such as utilization of health care and changes in medication) and they may have limited robustness. Also, patients may not be aware of whether medication changed over 1 year and they may be subject to memory bias. Also, more investigation into what role the group setting plays, would be interesting. Qualitative studies might be important in order to develop an understanding of the group-mechanisms and how the group may contribute to change.

## Conclusions

The concentrated micro-choice based treatment approach for patients with depression and/or anxiety yielded impressive and surprising results, with high acceptability, high levels of patient satisfaction and significantly improved levels of functioning starting at 3-month follow-up and maintained 12 months after treatment. Furthermore, reduction in the relevant symptoms were observed as early as 7 days after treatment and were retained at 12 months. We conclude that these preliminary findings are highly promising for concentrated transdiagnostic and cross-disciplinary micro-choice based group treatment for patients with depression and with anxiety and warrant further exploration.

## Data Availability

The data that support the findings of this study are available from Youwell A/S and Checkware, but restrictions apply to the availability of these data, which were used under license for the current study, and so are not publicly available. Data are however available from the authors upon reasonable request and with permission of Youwell A/S and Checkware. To request data, please contact Ane Wilhelmsen-Langeland, Bjørgvin DPS, Division of Psychiatry, Haukeland University Hospital, e-mail: anewil@ihelse.net, phone: +47 92843253.

## Abbreviations

PUSH: Project development of smarter health services
HiH: Helse i hardanger
MINI: Mini international neuropsychiatric interview
WSAS: Work and social adjustment scale
PHQ9: Patient health questionnaire-9
GAD7: Generalized anxiety disorder
PSWQ: Penn state worry questioniire
CFQ: Chalder fatigue questionnaire
BIS: Bergen insomnia scale
BIPQ: Brief illness perception questionnaire
OCD: Obsessive compulsive disorder
UP: Unified protocol
SDP: single-disorder protocols
SD: Standard deviation
ES: Effect size
DSM-IV-TR: American psychiatric association diagnostic and statistical manual of mental disorders-IV-text revision
